# A Recommendation for Revised Dose Calibrator Measurement Procedures for ^89^Zr and ^124^I

**DOI:** 10.1371/journal.pone.0106868

**Published:** 2014-09-09

**Authors:** Bradley J. Beattie, Keith S. Pentlow, Joseph O'Donoghue, John L. Humm

**Affiliations:** Medical Physics, Memorial Sloan Kettering Cancer Center, New York, New York, United States of America; Genentech, United States of America

## Abstract

Because of their chemical properties and multiday half lives, iodine-124 and zirconium-89 are being used in a growing number of PET imaging studies. Some aspects of their quantitation, however, still need attention. For ^89^Zr the PET images should, in principle, be as quantitatively accurate as similarly reconstructed ^18^F measurements. We found, however, that images of a 20 cm well calibration phantom containing ^89^Zr underestimated the activity by approximately 10% relative to a dose calibrator measurement (Capintec CRC-15R) using a published calibration setting number of 465. PET images of ^124^I, in contrast, are complicated by the contribution of decays in cascade that add spurious coincident events to the PET data. When these cascade coincidences are properly accounted for, quantitatively accurate images should be possible. We found, however, that even with this correction we still encountered what appeared to be a large variability in the accuracy of the PET images when compared to dose calibrator measurements made using the calibration setting number, 570, recommended by Capintec. We derive new calibration setting numbers for ^89^Zr and ^124^I based on their 511 keV photon peaks as measured on an HPGe detector. The peaks were calibrated relative to an ^18^F standard, the activity level of which was precisely measured in a dose calibrator under well-defined measurement conditions. When measuring ^89^Zr on a Capintec CRC-15R we propose the use of calibration setting number 517. And for ^124^I, we recommend the use of a copper filter surrounding the sample and the use of calibration setting number 494. The new dose calibrator measurement procedures we propose will result in more consistent and accurate radioactivity measurements of ^89^Zr and ^124^I. These and other positron emitting radionuclides can be accurately calibrated relative to ^18^F based on measurements of their 511 keV peaks and knowledge of their relative positron abundances.

## Introduction

### Zirconium-89

Zirconium-89 is a positron emitting radiometal with a 3.27 day half-life and a mean positron energy of 396 keV. It also emits 909 keV cascade gamma rays but with sufficient delay that they do not cause spurious coincidences [1]. These properties, along with its tendency to residualize in cells, make ^89^Zr an increasingly popular choice as a radiolabel for PET imaging studies of in vivo antibody distribution.

In a conference proceedings in 2006 [2], Avila-Rodriguez et al. described using a calibration setting number of 465 for the Capintec CRC-15R. However, when we applied this calibration setting number in phantom measurements seeking to verify the quantitative accuracy of the PET scanning procedures, discrepancies between the observed activity concentration derived from the PET data and the expected values based on dose calibrator measurements were noted.

Earlier work by Verel et al. in 2003 [3] proposed a dose calibrator measurement for ^89^Zr that involved using the setting for ^54^Mn and multiplying the displayed activity by 0.67. This procedure is independent of the dose calibrator model used but because it involves two steps, may be overlooked by many investigators and radiopharmacists in deference to the more recent value proposed by Avila-Rodriguez. The manufacturer of the dose calibrator used in our studies, Capintec, does not make a recommendation for ^89^Zr but based on Capintec's recommendation for ^54^Mn and their description of the relationship between calibration setting numbers and the dose calibrator response (see equation 1 derived from the CRC-15R Owner's Manual [4]), the procedure described by Verel corresponds to the use of a calibration setting number of 504 on the CRC-15R. 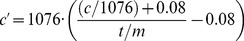
(1)


where: 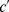
is the corrected calibration setting number, 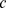 is the calibration setting number that was used (e.g. the one for ^54^Mn), 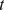 is the true activity and 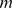 is the activity measured using calibration setting number 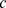 (note: 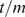 is 0.67 in the above).

Neither Verel nor Avila-Rodriguez, however, described in detail how the values they recommend were determined.

### Iodine-124

Iodine-124 is a radionuclide with a complex decay scheme including a 22.9% positron abundance and a 58% abundance of x-rays in the 20 to 40 keV range. Approximately half of its positrons are followed by prompt cascade 602 keV gamma-rays [5]. These cascades add spurious coincident events to the PET projection data, which if not properly corrected for, can lead to errors in the PET quantitation. The positrons and its 4.18 day half-life, make ^124^I an appealing isotope for use as a radiolabel in PET antibody studies and, in its iodide form, for PET-based dose estimates of radiotherapies involving [^131^I]-iodide.

In phantom studies involving ^124^I, we frequently noted discrepancies between PET derived activity concentrations and dose calibrator data, even though a correction for cascade coincidences was being used. Similar discrepancies were noted previously by Jentzen [6]. The problem appears to result from a combination of an inappropriate calibration setting number and the aforementioned x-rays. The x-rays may or may not be attenuated significantly depending upon the volume of the sample and what material the container is made of (this combination of properties here onward will be referred to as the measurement "geometry") thereby affecting the radioactivity measurement.

Capintec recommends using a calibration setting number of 570 for ^124^I on the CRC-15R [4] assuming a 5 mL solution in a 0.6 mm thick borosilicate glass vial. They warn, however, of an uncertainty of about +/-5% if a plastic or glass syringe, respectively, is used when measuring the activity. To avoid this uncertainty, we make use of the recommendation described by Wiarda in 1984 [7] to use a copper filter in order to remove the contribution of the ∼30 keV K x-rays from the dose calibrator measurement.

### Calibration

In the work we describe here, we utilize a dose calibrator measurement of an ^18^F sample made under precisely defined conditions as a reference standard against which we calibrate both our PET camera and our HPGe measurements. Our HPGe measurements of ^89^Zr and ^124^I, calibrated based on the 511 keV peak of the ^18^F reference, allowed us to determine precise calibration setting numbers to be used on the CRC-15R when measuring these radionuclides under various defined geometries. The dose calibrator we used was checked using a NIST traceable, ^18^F cross referenced, ^68^Ge/^68^Ga calibration standard (Radqual Model BM06S-681, serial # BM06068S14104102). This standard mimics a 5 mL syringe and can be hung from the dose calibrator's dipper. Daily accuracy tests of the dose calibrator established its stability over the time-course of all our measurements. PET measurements of phantoms containing ^89^Zr or ^124^I also served to cross-validate the new calibration setting numbers.

## Materials and Methods

### Radionuclides

The ^89^Zr and ^124^I sources used in these experiments were produced by the MSKCC Radiochemistry Core via the ^89^Y(*p*,*n*)^89^Zr and ^124^Te(p,n)^124^I reactions, respectively. Irradiations were conducted using an EBCO TR19/9 cyclotron (Ebco Industries Inc., Richmond, British Columbia, Canada). All measurements were conducted at least 5 days post end-of-bombardment. Radionuclidic purity was always greater than 99.98% as determined by gamma-spectroscopy using an HPGe detector (Canberra model GC2018) coupled to a calibrated multichannel analyzer (MCA, Canberra Inspector 2000, Canberra Industries, Oak Ridge, TN, USA). The MCA was calibrated by using ^133^Ba (81.0, 302.8 and 356.0 keV), ^109^Cd (88.0 keV), ^57^Co (122.1 keV), ^6^°Co (1173.2 and 1332.5 keV), ^137^Cs (661.6 keV) and ^22^Na (1274.5 keV) standard sources from Canberra Industries, Oak Ridge, TN, USA, and data were processed using the Genie-2000 software [8]. All ^18^F samples were purchased as clinical grade [^18^F]-FDG from IBA Molecular (Dulles, VA). Table 1 provides a summary of the properties of these three radionuclides, pertinent to the calculations in this paper.

**Table 1:** Radionuclide Properties.

Radionuclide	Half-life (hours)	Positron abundance (%)	Mean positron energy (keV)	Max positron energy (keV)	(20-200 keV) x-ray abundance (%)
^18^F	1.8	96.7	250	634	0
^89^Zr	78.4	22.7	396	902	0
^124^I	100.2	22.9	819	2140	58

Data in this table was taken from ICRP Publication 107 [11].

### Dose Calibrator

All dose calibrator measurements were made on a Capintec CRC-15R supplemented with a 4 cm lead Environmental Shield (item number 7300-2450, Capintec, Inc., Pittsburgh, PA) immediately surrounding the chamber. Backscatter from this shield while measuring ^18^F, ^89^Zr or ^124^I was determined to have negligible effect on the activity measurements by comparing shielded and unshielded measurements once for each radionuclide. Measurements were made with either the plastic dipper provided by the manufacturer or instead within a cylinder, 20 cm long and having a 4.1 cm outside diameter with 1.52 mm walls, of Type L rigid copper water pipe. The bottom half of the pipe was stuffed loosely with foam rubber to support the source at a location in the center of the measurement chamber. Foam extending out the bottom, centered the pipe within the well.

### HPGe Detector Setup

All calibration HPGe measurements were made with a Canberra HPGe detector (model GC2020, Canberra Industries, Inc. Meriden, CT) coupled to a calibrated multichannel analyzer (Canberra Inspector 2000). The GC2020 is a liquid nitrogen cooled Standard Electrode Coaxial Ge detector with a vertical slimline dipstick, 30 liter Dewar and endcap diameter of 7.6 cm. Its energy resolution (full width half max) at 122 and 1300 keV are 1.10 and 2.0 keV, respectively.

In all cases the source, consisting of a 10 mL solution in a 20 mL vial made of 1.13 mm thick borosilicate glass (typically used for liquid-scintillation counting and made by Kimble Chase, part #74504-20) surrounded by a 5mm thick polymethyl methacrylate cylinder, was suspended from the ceiling at a height of 1.68 m above the surface of the HPGe detector (oriented vertically). This large working distance was used for three reasons: 1) to maintain a low dead-time (< 10%); 2) to minimize the impact of small changes in this distance from sample placement to sample placement (variability judged to be less than 5 mm) and 3) to minimize pileup. The software controlling the Canberra HPGe detector measures counts as a function of a calibrated energy (i.e. a spectrum) and adjusts the actual time of the sampling to account for dead time.

### HPGe Data Processing

Immediately prior to and following the HPGe sample measurements on any given day, 5 minute background spectral measurements were made. For these measurements, all sources were removed from the room. The two background measures were compared to one another and examined for unexpected or interfering peaks caused by unseen sources introduced in the vicinity (e.g. on an adjacent floor). In all cases they were judged acceptable and averaged together to form a single background spectrum.

The HPGe spectral measurement of each ^18^F, ^89^Zr or ^124^I sample, was also acquired for 5 minutes. The averaged background spectrum was subtracted from each. The tails surrounding the 511 keV peak, 506-507 keV and 515-516 keV, in each background corrected spectral measurement were fitted with a ramp which was then subtracted from the peak. Note the full width half max resolution of the Doppler-broadened annihilation photopeak is about 2.6 keV. The remaining count data between 506.5 and 515.5 keV was numerically integrated and divided by the 5 minute target measurement time (thereby accounting for the dead time).

### ^18^F Reference Standard

Our calibration reference consisted of a 1 mL [^18^F]-FDG water solution in a 3 mL Beckton Dickinson plastic syringe (cat# 309657) with attached 19 gauge needle (Beckton Dickinson cat# 395186), suspended from the plastic dipper so as to be placed near the center of the chamber of a Capintec CRC-15R dose calibrator set on calibration setting number 484. In 2008, the US National Institute of Standards and Technology (NIST), in concert with Capintec, issued a recommendation to use a calibration setting number of 484 on the CRC-15R for this geometry in order to improve the accuracy of the ^18^F activity measurement [9]. After measurement on the dose calibrator, the contents of the syringe were transferred to a liquid scintillation vial (previously described) and the volume of the solution increased to 10 mL, rinsing the syringe into the vial in the process. Residuals were generally negligible but in any case measured and applied prior to the measurements designed to calibrate the HPGe detector setup. The HPGe measurements were made as described above. Factoring in the appropriate corrections for decay and positron abundance, the sensitivity of the HPGe detector setup, in units of integrated counts per positron, was determined.

### Sample Measurements

In total we measured 5 samples of ^89^Zr, 4 samples of ^124^I and for calibration purposes 6 samples of ^18^F in both the dose calibrator and on the HPGe detector. Measurements were made over a period of several weeks. On any given day in which a ^89^Zr or ^124^I measurement was made on the HPGe, a reference pair of calibration measurements using an ^18^F sample were made on the dose calibrator and on the HPGe to test for potential day-to-day differences in detector sensitivity.

The accuracy of the Capintec dose calibrator was evaluated using a NIST traceable ^68^Ge/^68^Ga positron standard that has been cross referenced to ^18^F. At the time of first measurement, this source contained 35.594 MBq ± 0.53% (95% confidence interval) of ^18^F equivalent radioactivity. Several measurements over a period of two weeks were made. Each of these measurements was found to be within 0.6% of the standard's nominal radioactivity level.

For most of the samples, only the liquid scintillation vial geometry was used. Measures of radioactivity using this geometry were calibrated based on a measurement at a single calibration setting number that was subsequently adjusted using equation 1. For a subset of the samples (one ^124^I and two ^89^Zr), dose calibrator measurements were made at each of six different geometries, a small volume in a 5 mL syringe (Beckton Dickinson cat# 309646) with attached needle (Beckton Dickinson cat# 395186), 3 mL in a 5 mL syringe with needle, and 10 mL in a glass liquid scintillation vial (Kimble Chase, part #74504-20), each with and without the copper filter. For each geometry five calibration setting numbers (-60, -30, +0, +30 and +60) bracketing what was thought to be approximately the correct number were used. In addition, for ^89^Zr the calibration setting numbers of 465 and 309 (the calibration setting number for ^54^Mn) were used. The activity measured at the 309 setting was subsequently multiplied by 0.67 as per Verel's recommendation. For ^124^I, a calibration setting number of 570 was also used.

The full bracketed measurements generally proceeded as follows. A small volume (typically below 0.1 mL depending on the stock concentration) of ^89^Zr or ^124^I was drawn into a 5 mL syringe. The activity of this sample varied between 10 and 40 MBq. The sample was then measured at each of the aforementioned calibration setting number settings with the syringe suspended from the ring on the plastic dipper. The time-of-day of each measurement was recorded. All measurements were then repeated, this time with the syringe centered within the copper filter (replacing the plastic dipper) at the center of the dose calibrator's chamber. Tap water was then drawn into the syringe to bring the volume up to 3 mL. Measurements with the plastic dipper and within the copper filter were once again repeated, each time incorporating adjustments to the central bracketed calibration setting number. Finally, the contents of the syringe were transferred to a Kimble Chase KG-33 borosilicate glass 20 mL scintillation vial (cat# 03-340-4C) and the volume of the solution brought up to 10 mL. Residual activity in the syringe was generally negligible but nevertheless was measured using the copper filter at one of the calibration setting number settings used previously. All radioactivity measurements were decay corrected to a single reference time. The residual, was expressed as a fraction of the total (determined at the same calibration setting number used in the copper filter measurement) and each of the activity measures was adjusted accordingly.

Plots of activity versus bracketed calibration setting number for each sample geometry were generated and least-squares fitted to a quadratic curve. The presumed "true" activity of each sample was determined by applying the HPGe sensitivity factor to the sample's integrated counts per second and accounting for positron abundance, decay and any residual losses. Using this activity, the quadratic associated with each measurement geometry was solved to determine the calibration setting number. The calibration setting numbers for the liquid scintillation vial, with and without the copper filter, were then used in a pair of additional cross-checking dose calibrator measurements. In all cases these measures confirmed the interpolated calibration setting number.

### PET Measurements

One of the ^89^Zr samples and one of the ^124^I samples, thus calibrated and residing in a liquid scintillation vial, was transferred (separately) to a water filled 20 cm diameter by 19 cm polymethyl methacrylate PET phantom. This phantom was then imaged on a General Electric Discovery STE PET/CT scanner. The ^89^Zr phantom was imaged while positioned in the center of the field of view while the ^124^I phantom was imaged both at the center and also displaced 9.3 cm vertically off-center.

This scanner uses BGO detectors and is capable of both 2D (with septa) and 3D (without septa) mode acquisitions. Its sensitivity at the center of the field of view was measured to be 2.2 cps/kBq in 2D and 8.6 cps/kBq in 3D. Its resolution in 2D is 5.4×5.4×5.4 mm at 1 cm from the central axis, falling off to 5.7×5.7×6.1 at 10 cm. In 3D at 1 cm it is 5.5×5.5×6.1 mm and at 10 cm it is 5.8×5.8×6.1 mm. The scatter fraction for the described 20 cm phantom when uniformly filled with ^18^F is estimated to be 18% in 2D mode, while in 3D it is 26%. The measurement of the ^89^Zr containing phantom was made in 3D mode while the measurements of ^124^I were made in 2D. The ^124^I raw data were corrected for cascade coincidences prior to image reconstruction using the convolution subtraction method described by Beattie et al. [10]. Volumes of interest were defined on each of the reconstructed images covering almost the entire interior volume of the phantom, but extending no closer than 1 cm to any surface. The mean activity concentration within this region, adjusted for decay and multiplied by the phantom volume, provided the PET estimate of the total radioactivity which was cross-checked against the HPGe and dose calibrator measurements.

It should be noted that particularly on some of the older PET scanners, it is wise to check that the positron abundance and decay rates entered into the scanner for ^89^Zr and ^124^I are indeed correct.

## Results

**Figure 1:**
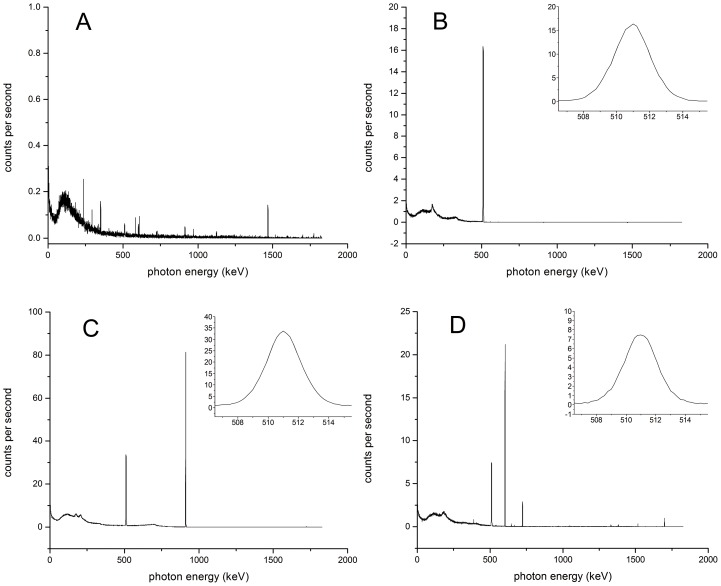
Photon energy spectra as measured by the Canberra HPGe detector for A) background, B) F-18, C) Zr-89 and D) I-124. Insets show close-up of 511 keV annihilation photon peak upon which calibrations were based.

Plots of background spectra measured on the HPGe showed negligible signal above baseline within the 511 keV window, 506-516 keV (see Figure 1A). The spectra for ^18^F, ^89^Zr and ^124^I (see Figure 1B, C and D, respectively) contained no significant peaks corresponding to radioactive impurities that might contribute to the signal measured at or near to the 511 keV peak.

The measures of the six ^18^F standards established the sensitivity of our HPGe setup to be 3.544e-05 integrated counts per positron. The coefficient of variation was 0.27%. No trend in this sensitivity was seen over time and no outliers were seen on any given day.

**Figure 2:**
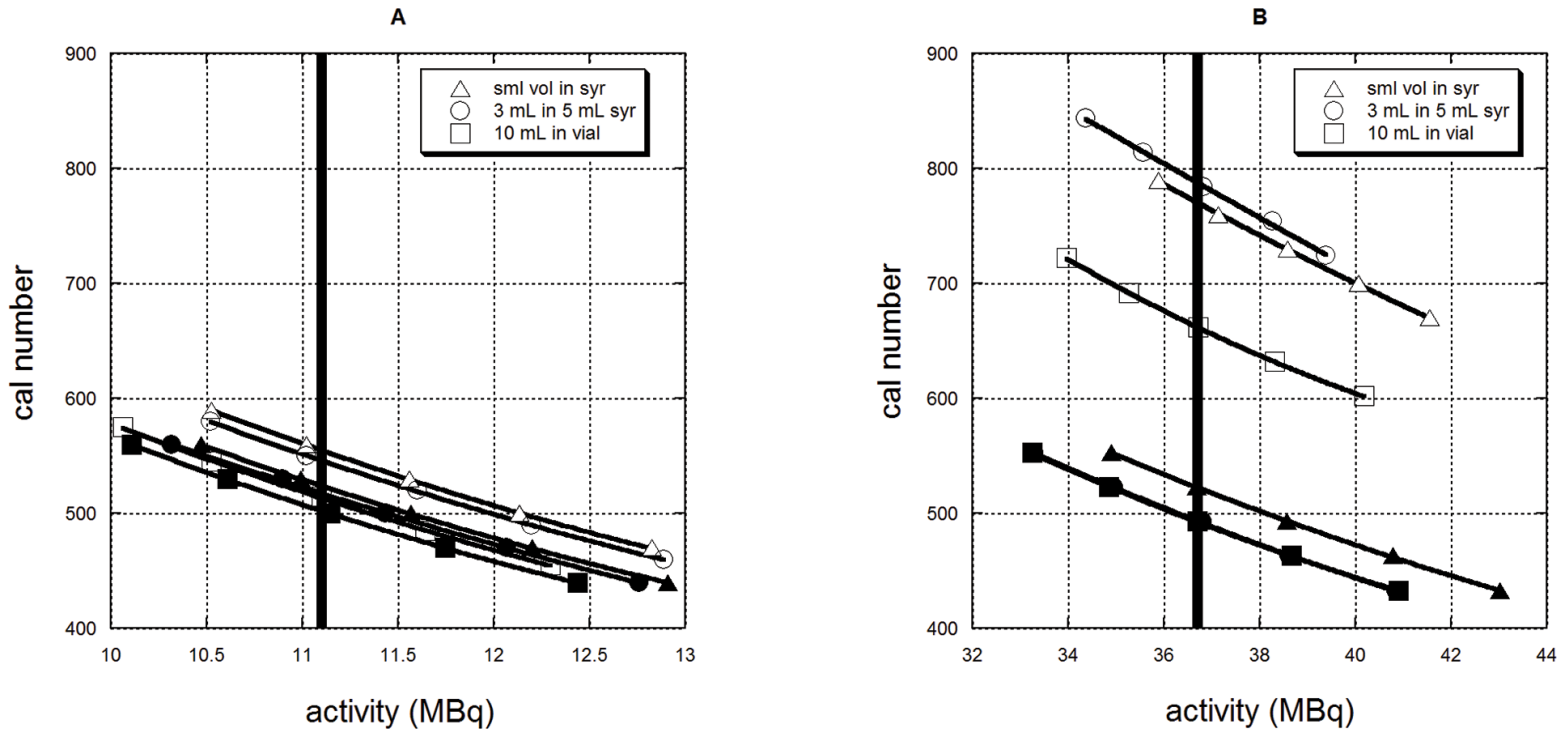
Activity versus calibration setting number as measured on a Capintec CRC-15R dose calibrator for A) Zr-89 and B) I-124, measured in different geometries. Filled symbols identify the curves corresponding to the measurements with the copper filter; open symbols without the filter. Triangles correspond to the small volume in the 5 mL syringe, circles to the 3 mL volume in the 5 mL syringe and squares to the 10 mL volume in the liquid scintillation vial. Note that for I-124 the filled squares almost completely obscure the filled circles.

Plots of activity versus calibration setting number for selected samples of ^89^Zr and ^124^I measured in various geometries are shown in Figures 2A and B, respectively. Groups of points corresponding to a given geometry are fitted with the quadratic curve shown. The vertical bars indicate the correct activity as determined by the HPGe measurement. The height at which this bar intersects the fitted curves describes the correct calibration setting number to use for the corresponding geometry and radionuclide.

As can be appreciated from the range of calibration setting numbers needed to correctly measure ^124^I when the copper filter is *not* used, the ^124^I measurements are very geometry sensitive. Conversely, use of the copper filter greatly reduces the range of calibration setting numbers needed to the point where a single average calibration setting number could be used without significant error, especially if the measurement of very small volumes is avoided.

The calibration setting numbers for the syringe measurements (columns 2 and 3) in Table 2 have been interpolated from the fitted curves shown in Figure 2. The calibration setting numbers for the liquid scintillation vial (column 4) have been averaged over all calibrations. The coefficient of variation is also shown. When bracketed measures were made, the calibrated calibration setting number was determined by interpolation of the fitted quadratic. When a single measure using a single calibration setting number was made, the corrected calibration setting number was arrived at using equation 1. Our recommendation of 517 as the calibration setting number to use for ^89^Zr is the average of the 3 ml in a 5 mL syringe and liquid scintillation vial calibration setting numbers.

**Table 2:** Recommended Calibration setting Numbers.

	Small vol 5 mL syringe calibration setting number	3 mL in 5 mL syringe calibration setting number	10 mL in liq scint vial calibration setting number +/- coef_of_var
^89^Zr	529	524	510 +/- 0.3%
^89^Zr in Cu filter	500	497	498 +/- 0.2%
^124^I	749	788	664 +/- 0.5%
^124^I in Cu filter	507	494	494 +/- 0.9%

Table 3 shows the errors that would have been encountered if we had assumed that the standard calibration setting numbers for ^89^Zr and ^124^I were correct. For ^89^Zr, these results corroborate the procedure proposed by Verel and suggest a small but significant improvement over using a calibration setting number of 465. In general we found activity measurements of very small volumes to be relatively inaccurate. We speculate that for these small volumes, a relatively large fraction of the volume is within or near to the metal syringe needle and thus subject to different photon attenuation and positron stopping potential.

**Table 3:** Error in Radioactivity when Standard Calibration Setting Numbers are Used.

	Small vol 5 mL syringe (% error)	3 mL in 5 mL syringe (% error)	10 mL in liq scint vial (% error)
^89^Zr at 465	+12.0	+11.0	+8.00
^89^Zr at 309 × 0.67	+5.1	+3.7	+1.1
^124^I at 570	+28.0	+33.6	+14.9

The PET measurements of ^89^Zr confirm the HPGe calibrations to within 1.11%. For ^124^I, the PET measurements agreed with the HPGe to within 0.43% when the phantom was centered in the field of view and to within 1.05% when the phantom was off-center.

## Discussion

The absolute accuracy of the calibration setting numbers we propose for ^89^Zr and ^124^I are ultimately dependent upon the accuracy of our dose calibrator measurement of ^18^F. To guard against the potential that our absolute quantitation was off, we used a well-defined geometry when making the ^18^F activity measurements, one that precisely mimicked the measurement conditions that were used by NIST in 2009. We guarded against the potential that our dose calibrator was miscalibrated by calibrating it against a NIST traceable, ^18^F cross referenced, ^68^Ge/^68^Ga dose calibrator reference standard.

The remaining potential source of error in our calibration measurements is related to the difference in the positron energies of ^18^F, ^89^Zr and ^124^I. Higher energy positrons are more likely to escape the liquid scintillation vial, annihilate and produce 511 keV photons remote from the vial. The positrons of ^18^F have a mean energy of 250 keV and max of 634 keV, ^89^Zr has a mean of 396 keV and max of 902 keV, while ^124^I has positrons with a mean energy of 819 keV and max of 2.14 MeV [11].

We minimized the impact of these positron energy differences by ensuring that the overwhelming majority of the positrons would annihilate within or very near to the container holding the radioactive sample. The relatively large (10 mL) volume of the radioactive solution has a small surface area to volume ratio. The glass of the liquid scintillation vial and the thick additional surrounding plastic, further ensure that the positrons are stopped locally. Based on the NIST ESTAR Stopping Power and Range Tables for Electrons [12] and on the positron energy spectra available through the DECDATA software [13], we calculated that, of the positrons at the interior surface of the glass vial emitted outward perpendicular to the surface, just 4.5% of the ^124^I, 0.2% of the ^89^Zr and 0.007% of the ^18^F escape past the cylinder. Considering the small fraction of positrons at the glass surface and directed outward, the overall fraction of lost positrons is negligibly small.

In theory, a similar positron energy dependent difference exists for the PET measurements as well, but here with a PET phantom volume over 500 times larger, the effect is clearly negligible. That the PET measures corroborate the HPGe measurements is a further indication that the effect is indeed negligible in both circumstances.

The confidence in the corroboration between the PET and HPGe measurements is strongest between ^89^Zr and ^18^F, both because the positron energy distributions are more similar, but also because the PET measures are more similar in that neither ^89^Zr nor ^18^F require a correction for cascade coincidences. Although ^89^Zr does indeed have 909 keV gamma-ray emissions that are in cascade with its positron, the half-time of the intermediate state (16 seconds) is longer than the timing window defining coincidences in PET.

^124^I, on the other hand, does suffer from spurious cascade coincidences. We chose to acquire our ^124^I PET data in 2D mode so as to minimize the magnitude of this confound, however high quantitative accuracy still requires the application of a cascade coincidence correction. The correction procedure we chose to apply is based on first principles, not a heuristic designed to produce an expected (and potentially erroneous) quantitative outcome.

The net result of our work with regard to ^89^Zr is a recommendation to use a calibration setting number that more closely agrees with measurement procedure published by Verel. Our one-step measurement is slightly easier than Verel's but more importantly our results select between the two published (and significantly different) calibrations, the second being the 465 calibration setting number recommended by Avila-Rodriguez. Our work also shows the degree to which a single calibration setting number is accurate for ^89^Zr over a range of geometries.

**Figure 3:**
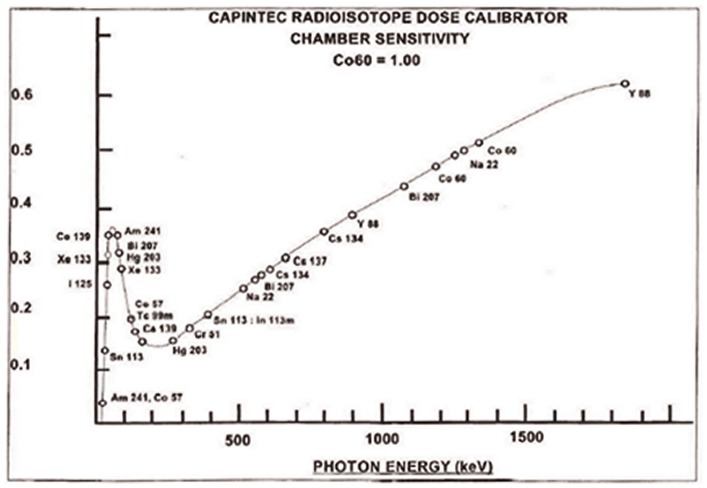
Plot of photon energy versus sensitivity for a Capintec CRC-15R dose calibrator. (Reprinted with permission of Capintec Inc. from the CRC-15R Owner's Manual).

In the case of ^124^I, we show that using the Capintec recommended calibration setting number results in large errors. There is also great variability depending on the volume and container material used in the measurement. As a means of avoiding these errors, we recommend the use of a copper filter and a calibration setting number of 494 regardless of the container and volume. The reason the copper filter has this effect is because it removes the contribution of the x-rays emitted by ^124^I in the 20-40 keV range from the dose calibrator measurement. The x-rays in this range (in total) are 58% abundant [11] but in terms of total energy output from ^124^I, their contribution is very small. However, for many dose calibrators the contribution of a 40 keV photon can be as great or greater than a photon with 10 times that energy (see Figure 3). Without the copper filter, the attenuation of the 20-40 keV x-rays is very variable, depending on the path length through water, plastic and glass in the sample. The photoelectric plus Compton scattering attenuation coefficient for a 40 keV photon is just 0.24 cm^-1^ in water, 0.25 cm^-1^ in plastic and 0.88 cm^-1^ in glass, whereas the attenuation coefficient in copper is 43 cm^-1^ [14]. Thus even a small amount of copper removes virtually all of these photons (the 1.52 mm copper filter we used removes 99.9%) while allowing most of the higher energy gamma-rays to still pass through. The net result is a more robust but somewhat less sensitive measurement of ^124^I activity.

^89^Zr also has a significant abundance of x-rays at energies that could conceivably cause geometry dependent variability in a dose calibrator measurement. These photons, in fact, likely explain the slightly reduced variability in our copper filtered measurements relative to the unfiltered. These x-rays however are in the 13 to 15 keV range which although technically above Capintec's stated 13 keV threshold, contribute much less to the radioactivity measurement compared to the 20 to 40 keV x-rays of ^124^I. For most purposes, measurements of ^89^Zr without a copper filter should be sufficiently accurate. However, investigators seeking greater accuracy are urged to use the copper filter with ^89^Zr at a calibration number setting of 498.

Our goal in proposing these new calibration number settings and measurement procedures for ^89^Zr and ^124^I is to improve the accuracy of radioactivity measurements involving these radionuclides sufficient for their predominant use, in PET imaging studies. We urge others to make more careful measurements and propose calibration number settings for specific geometries to be used in applications requiring greater accuracy and precision.

## Conclusion

Based on this work, we propose a new calibration setting number, 517, to be used on a Capintec CRC-15R dose calibrator when measuring samples of ^89^Zr. Use of this number will result in approximately a 10% change in the activity measurement compared to measurements made with another published and widely used calibration setting number of 465. Our value is relatively close to an alternate two-step procedure, confirming the accuracy of that work.

We also propose the use of a copper filter and corresponding new calibration setting number, 494, to be used in activity measurements of ^124^I. Use of this filter will avoid geometry dependent errors in the ^124^I activity measurement. Continued use of the Capintec recommended setting of 570 can result in overestimates of the radioactivity as high as 33%. Errors of this magnitude may have serious consequences for patients if the information is used to determine the activity to be administered for therapeutic purposes and may need to be reported as misadministrations in some jurisdictions.

For each of the radionuclides, ^18^F, ^89^Zr and ^124^I, with or without the copper filter, we found that very small volumes in the 5 mL syringe were measured less accurately in the dose calibrator at the recommended settings. Therefore we suggest either avoiding this geometry when accurate radioactivity measurements are needed, or that individual users derive their own calibration number settings for this geometry.

## Acknowledgments

The authors wish to thank Dr. Serge Lyashchenko, Charles Davis and Yiauchung Sheh for providing us with radionuclide and access to their facilities. We further acknowledge the Radiochemistry & Molecular Imaging Probes Core of MSKCC. We wish to thank Capintec Inc. for allowing us to reprint the graph in Figure 3. And finally we wish to make note that CRC is a registered trade name of Capintec.

## Supporting Information

Table S1 **Zr-89 Bracketed dose calibrator measurements.**
(XLSX)

Table S2 **I-124 Bracketed dose calibrator measurements.**
(XLSX)

Table S3 **HPGe spectra and PET measurements.**
(XLSX)
